# Recent Patterns in Population-Based HIV Prevalence in Swaziland

**DOI:** 10.1371/journal.pone.0077101

**Published:** 2013-10-15

**Authors:** George T. Bicego, Rejoice Nkambule, Ingrid Peterson, Jason Reed, Deborah Donnell, Henry Ginindza, Yen T. Duong, Hetal Patel, Naomi Bock, Neena Philip, Cherry Mao, Jessica Justman

**Affiliations:** 1 Centers for Disease Control and Prevention (CDC), Mbabane, Swaziland; 2 Ministry of Health, Mbabane, Swaziland; 3 International Center for AIDS Care and Treatment Programs (ICAP), Columbia University, Mbabane, Swaziland; 4 Fred Hutchinson Cancer Research Center, Seattle, Washington, United States of America; 5 CDC Atlanta, Division of Global HIV/AIDS, Atlanta, Georgia, United States of America; 6 ICAP, Columbia University, New York, New York, United States of America; Kliniken der Stadt Köln gGmbH, Germany

## Abstract

**Background:**

The 2011 Swaziland HIV Incidence Measurement Survey (SHIMS) was conducted as part of a national study to evaluate the scale up of key HIV prevention programs.

**Methods:**

From a randomly selected sample of all Swazi households, all women and men aged 18-49 were considered eligible, and all consenting adults were enrolled and received HIV testing and counseling. In this analysis, population-based measures of HIV prevalence were produced and compared against similarly measured HIV prevalence estimates from the 2006-7 Swaziland Demographic and Health. Also, measures of HIV service utilization in both HIV infected and uninfected populations were documented and discussed.

**Results:**

HIV prevalence among adults aged 18-49 has remained unchanged between 2006-2011 at 31-32%, with substantial differences in current prevalence between women (39%) and men (24%). In both men and women, between since 2006-7 and 2011, prevalence has fallen in the young age groups and risen in the older age groups. Over a third (38%) of the HIV-infected population was unaware of their infection status, and this differed markedly between men (50%) and women (31%). Of those aware of their HIV-positive status, a higher percentage of men (63%) than women (49%) reported ART use.

**Conclusions:**

While overall HIV prevalence remains roughly constant, age-specific changes strongly suggest both improved survival of the HIV-infected and a reduction in new HIV infections. Awareness of HIV status and entry into ART services has improved in recent years but remains too low. This study identifies opportunities to improve both HIV preventive and care services in Swaziland.

## Introduction

HIV is the leading public health concern in Swaziland. Swaziland’s Demographic and Health Survey (SDHS), conducted in 2006-7, demonstrated a generalized epidemic with an HIV prevalence of 26% among of 15-49 year olds [1]. Faced with the highest HIV prevalence rate in the world, the Swaziland Ministry of Health (MOH) has expanded access to key HIV services: HIV testing and counseling, HIV care and antiretroviral therapy (ART), and prophylaxis for the prevention of mother-to-child transmission (PMTCT) of HIV [2]. In 2011 based on randomized control trials showing the potent effect of medical male circumcision (MMC) in reducing risk of HIV acquisition from heterosexual exposure in men [3,4,5], the MOH launched a national campaign to increase the uptake of voluntary MMC among HIV-uninfected men ages 15-49. The current study, the Swaziland HIV Incidence Measurement Survey (SHIMS), was designed to assess the impact of this national combination HIV prevention strategy on HIV incidence in Swaziland. In this paper, we describe the cross-sectional (2011) estimates of HIV prevalence as well as HIV testing history and ART use among the Swaziland population and compare these estimates from SHIMS with data from the 2006-07 SDHS to assess trends. 

## Methods

### Study design

The overall design of SHIMS was based on the direct measurement of HIV incidence at the population level before and after the scale-up of the national combination HIV prevention strategy. To identify eligible participants for the first incidence cohort, a nationally representative, household-based cross-sectional survey was conducted from December 2010 to June 2011, with key epidemiologic and bio-demographic information collected from Swaziland adults, 18 to 49 years of age. At ages 50+ years, recent (incident) HIV infections are very infrequent. 

The household sample size was calculated at 14,927 to permit 80% power to detect a 45% reduction in HIV incidence in men from pre- to post- national scale up of the national combination HIV prevention strategy, assuming a baseline HIV incidence of 2.0% among men. We assumed a design effect of 1.25 (based on previous similar DHS surveys), a retention rate of 90%, a household vacancy rate of 13% and a household refusal rate of 5%; and that 10% of men would not be contactable, 23% would be HIV infected and 5% would refuse to participate in the survey. The households were selected using a two-stage cluster sampling procedure, similar in design to that used by the SDHS. In the first stage, a systematic random sample of 575 of the country’s 2076 enumeration areas (EAs) was drawn. In the second stage, a full listing of all households was conducted within each of the 575 EAs. This household listing provided the sampling frame from which a random sample of 26 households was drawn from each EA. Field teams contacted the selected households, and conducted a census of household residents with the head of household (or other available responsible adult household member). 

Survey eligibility criteria included residing in or sleeping in the household the night before, reporting an age between 18-49 years inclusive, and the ability to provide informed consent in either SiSwati or English. Further information contributing to the SHIMS sampling methodology and weighting are provided elsewhere [6].

### Data Collection and Analysis

Interviewers administered questionnaires in SiSwati or English to survey participants on demographic, clinical and behavioral topics, including self-reported male circumcision status. HIV testing, including pre- and post-test counseling, was conducted in a private location in or just outside of the home. Blood samples were collected by venipuncture and rapid HIV testing was performed using *Determine*™ HIV-1/2Ag/Ab Combo (Alere, Japan). *Determine* reactive samples were confirmed with Uni-Gold™ HIV Test (Trinity Biotech, Ireland), following the country’s serial testing algorithm. Internal and external quality assurance procedures were implemented throughout the survey. Indeterminate test results were resolved by a two HIV EIA testing algorithm (Genscreen HIV-1/2 Version 2 Assay, Bio-Rad Laboratories, California, USA; and Vironostika HIV Uni-Form II plus O, bioMerieux Inc., France), and final HIV status based on the EIA results were returned to participants in the context of high quality post-test counseling including referral to prevention and, if indicated, HIV care services (pre-ART, CD4+ testing). 

Survey weights were applied to the data, accounting for differential probability of selection in the cluster sampling procedure and survey non-response rates. Given N’s (with the exception of Table 1) are estimated for SHIMS-sized population. HIV prevalence was based on testing positive in the survey; self-reported HIV status was not used. Illustrations of a circumcised and uncircumcised penis were provided to assist participants in correctly identifying their own (men) or their partner’s (women) circumcision status. Analyses were performed with SAS version 9.2 (SAS Institute, Cary, NC).

**Table 1:** Household Response Rates and Individual Response Rates.

	**Residence**		
	**Urban**	**Rural**	**Total**
**Households**			
Households selected	4,208	10,683	14,891
Households not occupied during survey period	472	1,084	1,556
Households occupied	3,710	9,599	13,335
Households interviewed	3,523	9,048	12,571
**Household response rate (%)**	**94.3 %**	**94.3 %**	**94.3 %**
**Women age 18-49**			
Number of eligible women	3,759	9,765	13,508
Number of eligible women interviewed	3,195	7,847	11,042
**Eligible women response rate (%)**	**85.0 %**	**80.0 %**	**81.3 %**
**Men age 18-49**			
Number of eligible men	3,028	8,020	11,048
Number of eligible men interviewed	2,066	5,064	7,130
**Eligible men response rate (%)**	**68.2 %**	**63.1 %**	**64.5 %**
**Total number of individuals in survey**	**5,261**	**12,911**	**18,172**

### Ethical considerations

All study participants provided written informed consent prior to the collection of data and blood samples. Ethical approval was obtained from the Swaziland Scientific and Ethics Committee, Columbia University Institutional Review Board (IRB) and U.S. Centers for Disease Control and Prevention IRB before initiation of field work. 

## Results

### Response Rates

The 14,891 selected households were approached, 13,335 were found occupied, and of these, 12,571 households participated in the baseline cross-sectional survey, resulting in a household response rate of 94.3% (Table 1). Urban and rural household response rates did not differ substantially. Among participating households, 13,508 women and 11,048 men aged 18-49 were identified as eligible. Despite up to 9 repeated visits to obtain individual interviews, some eligible person were either not contacted or when contacted refused to participate or make themselves available for interview. Eighty-one (81) % of women (11,042) and 65% of men (7,130) took part in the survey, having agreed to both the HIV test and a face-to-face interview. Urban men had a higher response rate than their rural counterparts (68% versus 63%). No significant differences in response rates were observed by age category or region (data not shown). 

### Socio-demographic Characteristics of the Study Population

Over half (56%) of the population was under age 30 and most (70%) lived in rural areas of the country (Table 2), a finding which mirrors the population distribution reflected in the most recent National Census (2007) [7]. Over a quarter (29%) of adults reported ever attending only primary school, 50% reported attending secondary school, and 14% reported attending tertiary education. Seven percent of participants reported never attending school. Education-related differentials between surveyed men and women were minimal. 

**Table 2:** Socio-demographic characteristics of SHIMS participants (ages 18-49).

	**Men (%**)	**Women (%)**	**Total (%**)
**Age**			
18-19	11.9	10.1	10.9
20-24	25.1	25.3	25.2
25-29	20.2	19.5	19.8
30-34	15.2	13.8	14.5
35-39	11.9	12.3	12.1
40-44	8.7	9.9	9.4
45-49	6.8	9.1	8.1
**Residence**			
Urban	29.5	30.1	29.9
Rural	70.5	69.9	70.1
**Region**			
Hhohho	28.6	28.4	28.5
Manzini	32.9	34.0	33.5
Shiselweni	17.8	18.8	18.3
Lubombo	20.6	18.7	19.6
**Education^1^**			
Did not attend	6.5	6.4	6.5
Primary	28.1	29.6	28.9
Secondary	48.8	50.8	49.9
Tertiary	16.2	12.7	14.3
**Current marital status**			
Not married, never had sex	15.8	5.7	10.3
Not married, ever had sex	44.5	41.7	43.0
Married, living with partner	25.4	28.6	27.1
Married, partner stays elsewhere	10.3	19.9	15.5
Married, unknown living situation	0.7	2.5	1.7
**No. Sexual partners last 6 months**			
0	27.2	18.2	22.3
1	53.6	77.4	66.5
2 or more	18.2	3.8	10.4
**Currently Pregnant?**			
Yes		7.0	
No		93.0	
**Circumcision status**			
Circumcised	17.1		
Not Circumcised	82.9		
**TOTAL (%)**	100.0	100.0	100.0

^1^ Refers to highest level of education ever attended, whether or not that level was completed

Nearly one-half of women and nearly two-thirds of men age 18-49 reported not being married, and more unmarried men than women reported never having had sex (16 % versus 6%). More women (22%) than men (11%) reported having a marital partner who either lived in a different household or whose living/sleeping arrangements were unknown. Seven (7) percent of women reported being pregnant.

Overall, 22% of participants reported having no sexual partners in the prior six months, 27% of men and 18% of women. Two-thirds (67%) reported having one partner in the past 6 months, 54% of men and 78% of women; while 10% of participants reported having two or more partners, 18% of men and 4% of women. One in 6 (17%) of men reported that they were circumcised.

### HIV Prevalence: Levels and Trends

**Figure 1:**
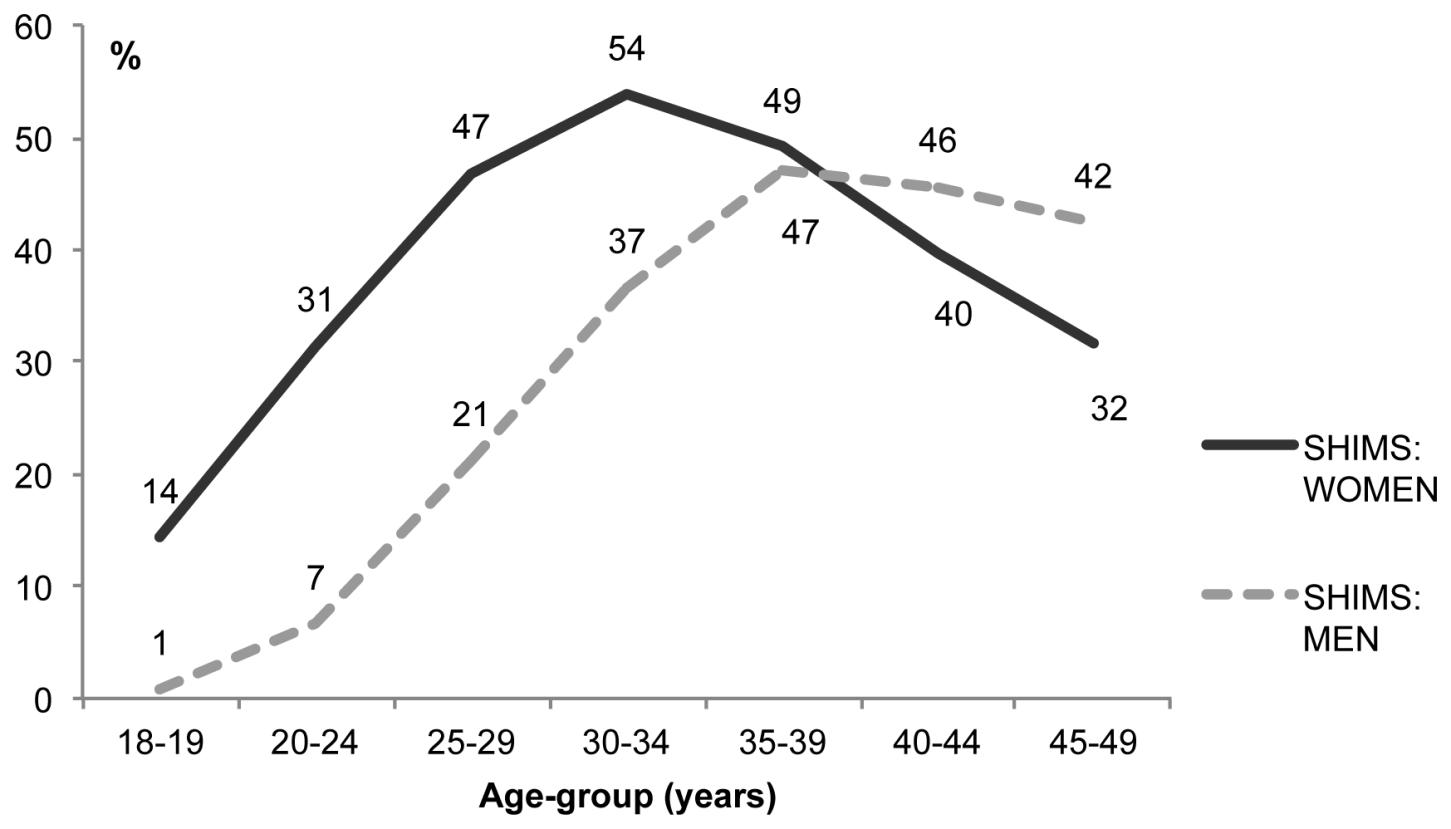
HIV prevalence (%) by age group and sex. SHIMS 2011.

HIV prevalence was 32% among adults aged 18-49 years (Table 3), 24% among men and 39% among women. In both men and women, HIV prevalence increased steeply with age and peaked before age 40 (Figure 1). Peak HIV prevalence was lower for men (47%) than women (54%) and occurred at an older age (35-39 versus 30-34). The difference in HIV prevalence by sex was most pronounced among young adults: HIV prevalence among <25 year olds was five times higher among women than men (26% versus 5%), a pattern commonly observed in sub-Saharan Africa associated with earlier age at first sex and exposure to HIV in women.

**Table 3:** HIV prevalence among adults (ages 18-49) in Swaziland, by selected socio-demographic characteristics.

		**Women**		**Men**		**Total**
	**%**	**N Tested**	**%**	**N Tested**	**%**	**N (combined sexes)**
**Age**						
18-19	14.3	992	0.8	995	7.6	1987
20-24	31.5	2489	6.6	2093	20.1	4582
25-29	46.7	1923	21.3	1682	34.9	3605
30-34	53.8	1361	36.6	1267	45.5	2629
35-39	49.1	1209	47.0	993	48.2	2202
40-44	39.7	975	45.5	728	42.2	1703
45-49	31.6	894	42.5	570	35.8	1465
**Residence**						
Urban	38.7	2965	24.5	2460	32.3	5425
Rural	38.9	6879	23.9	5869	32.0	12,747
**Region**						
Hhohho	37.4	2799	23.3	2385	30.9	5183
Manzini	40.4	3348	25.3	2743	33.6	6091
Shiselweni	37.5	1851	22.3	1482	30.7	3333
Lubombo	39.4	1845	24.9	1719	32.4	3565
**Education^1^**						
Did not attend	48.2	633	40.8	540	44.8	1173
Primary	45.9	2912	31.3	2335	39.4	5246
Secondary	36.8	5000	20.6	4067	29.6	9067
Tertiary	25.6	1253	15.5	1349	20.3	2602
**Current marital status**						
Not married, never had sex	3.6	554	0.9	1320	1.7	1875
Not married, ever had sex	43.2	4104	20.7	3707	32.5	7810
Married, living with partner	37.2	2811	38.7	2112	37.8	4923
Married, partner stays elsewhere	39.0	1956	36.2	854	38.2	2810
Married, unknown living situation	59.5	250	54.5	57	58.6	307
**No. partners (past 6 months)**						
0	33.8	1794	8.7	2267	19.8	4062
1	39.2	7618	30.6	4466	36.0	12,083
2 or more	54.5	373	28.1	1515	33.3	1887
**Currently pregnant**						
Yes	37.9	669				
No	38.8	8864				
**Circumcision status**						
Circumcised			15.7	1373		
Uncircumcised			25.7	6633		
**TOTAL NUMBER OF PARTICIPANTS**	**38.8**	**9843**	**24.1**	**8329**	**32.1**	**18,172**

**SHIMS 2011.**

^1^ Refers to highest level of education ever attended, whether or not that level was completed

**Figure 2:**
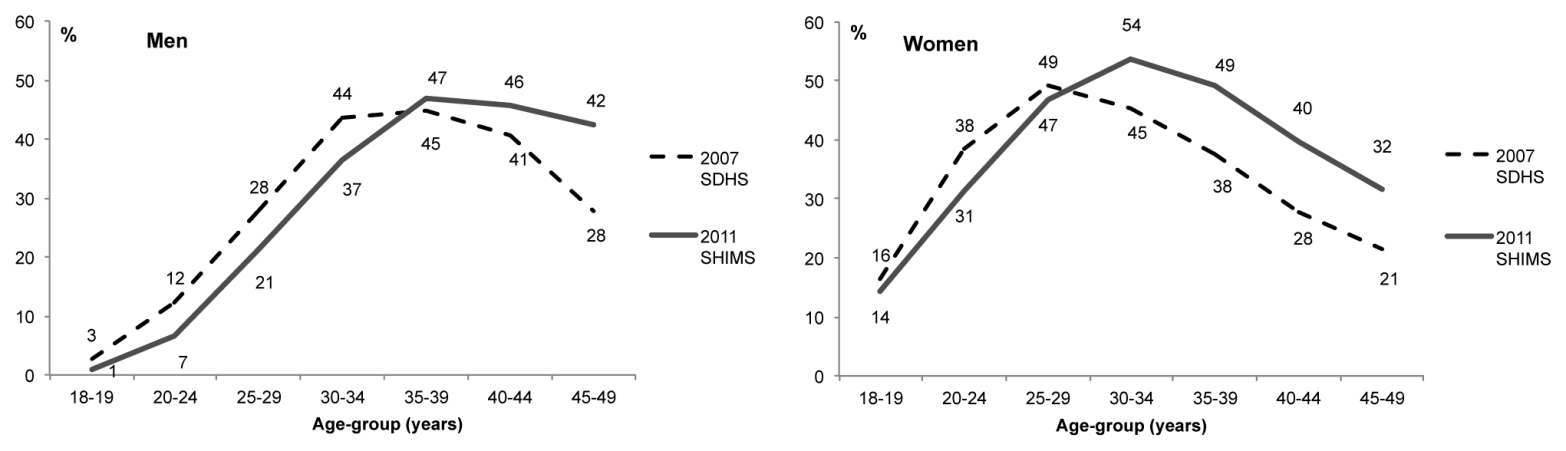
HIV prevalence (%) in men and women, by age group and survey. 2006-2007 SDHS and 2011 SHIMS.

The data from the current study allow a comparison of HIV prevalence rates and trends with data from the 2006-2007 Swaziland DHS study. The two surveys were very similar, with the SDHS based on a representative sample of 275 EAs and a response rate of 94.8% [1], similar to the SHIMS response rate of 94.4%. The 2006-2007 SDHS HIV prevalence, recalculated for 18-49 year olds was 31%, indicating that by this aggregate measure, the HIV epidemic in Swaziland has shown little change between the between 2007 and 2011. Figure 2 describes inter-survey changes in the age-pattern of HIV prevalence in men and women. While overall HIV prevalence (males and females combined, 18-49) was nearly the same in both surveys (31-32%), important changes in HIV prevalence are evidenced in age and sex decompositions. First, in both men and women, from the 2006 SDHS to SHIMS 2011, there have been declines in HIV prevalence at each age up through age 25-29 for women and 30-34 for men. The change is especially evident in men. Next, in both sexes, peak prevalence is higher and occurs at older ages in SHIMS 2011 compared to 2006-2007 SDHS. The pattern is especially evident in women with peak prevalence increasing from 49% at 25-29 years to 54% at 30-34 years old. Lastly, HIV prevalence remains higher at all ages after the peak in SHIMS 2011 compared to 2006-2007 SDHS; this pattern again is more pronounced in women.

### HIV Prevalence by Socio-demographic characteristics

HIV prevalence was similar in urban and rural areas, and across the four regions of Swaziland, ranging from 31% in Shiselweni and Hhohho to 34% in Manzini (Table 3). HIV prevalence varied substantially by education attainment, with higher HIV prevalence observed among both men and women with less education. In men, HIV prevalence among non-school attenders was more than double (41% versus 16%) that of those who attended a tertiary institution (college, university).

Unmarried men and women who reported never having had sex had relatively low HIV prevalence rates (4% women, 1% men). Infections in men and women reporting having had no previous sexual activity is likely due to underreporting associated with prevailing social unacceptability of early sexual debut, especially among girls. Another possible explanation is that coerced sex and rape was not understood and reported as sexual activity. The prevalence of HIV was substantially higher in sexually active unmarried women (43%) compared to sexually active unmarried men (20%). HIV prevalence among women did not vary by pregnancy status (38% for pregnant; 39% for non-pregnant). Among married men and women, HIV prevalence varied little by whether living with partner or not. In both women and men, higher reported numbers of sexual partners (last 6 months) were associated with higher HIV prevalence. In men, HIV prevalence was markedly higher among those reporting one partner in the last six months compared to those reporting no partners (31% versus 9%). There was however little difference in HIV prevalence between men reporting one versus two or more partners (31% versus 28%). Uncircumcised men had a 26 percent HIV prevalence rate compared to 16 percent among circumcised men, although this can be explained in part by the preponderance of circumcised men being younger (i.e. less cumulative exposure to HIV risk). 

### HIV Testing History, Awareness of Serostatus and ART Uptake

Overall, 71% of adults in Swaziland reported having received HIV testing services in the past (Table 4). More women (84%) than men (55%) reported prior HIV testing, regardless of current serostatus. Among all HIV-infected adults, 62% were already aware of their positive serostatus while 38% were unaware, either because they had not tested previously (17%) or because they did not receive a positive result in an earlier test (21%). 

**Table 4:** HIV testing history and current HIV status, among adults aged 18-49, men and women^1^.

	**Women**						**Men**						**Total**					
	**HIV+**		**HIV-**		**Total**		**HIV+**		**HIV-**		**Total**		**HIV+**		**HIV-**		**Total**	
**Self-reported HIV testing prior to SHIMS^2^**	**N**	**%**	**N**	**%**	**N**	**%**	**N**	**%**	**N**	**%**	**N**	**%**	**N**	**%**	**N**	**%**	**N**	**%**
Never had HIV test	364	**9.7**	1178	**19.6**	1542	**15.8**	597	**30.3**	3136	**49.9**	3733	**45.2**	961	**16.7**	4314	**35.1**	5275	**29.3**
Ever had HIV test - Positive	2590	**68.7**	21	**0.3**	2611	**26.7**	995	**50.4**	8	**0.1**	1003	**12.1**	3584	**62.4**	28	**0.2**	3612	**20.0**
- Negative	674	**17.9**	4702	**78.5**	5376	**55.1**	291	**14.8**	2977	**47.4**	3268	**39.6**	965	**16.8**	7679	**62.6**	8644	**48.0**
- Indeterminant/Unknown	142	**3.8**	94	**1.6**	236	**2.4**	89	**4.5**	161	**2.6**	250	**3.0**	231	**4.0**	255	**2.1**	486	**2.7**
**Total**	**3770**	**100**	**5994**	**100**	**9764**	**100**	**1972**	**100**	**6282**	**100**	**8254**	**100**	**5740**	**100**	**12276**	**100**	**18017**	**100**

SHIMS 2011.

^1^ Restricted to people who tested and received results in SHIMS

^2^ # missing self-reported HIV testing data in SHIMS: 51 HIV+ women, 29 HIV- women; 36 HIV+ men, 39 HIV- men.

**Figure 3:**
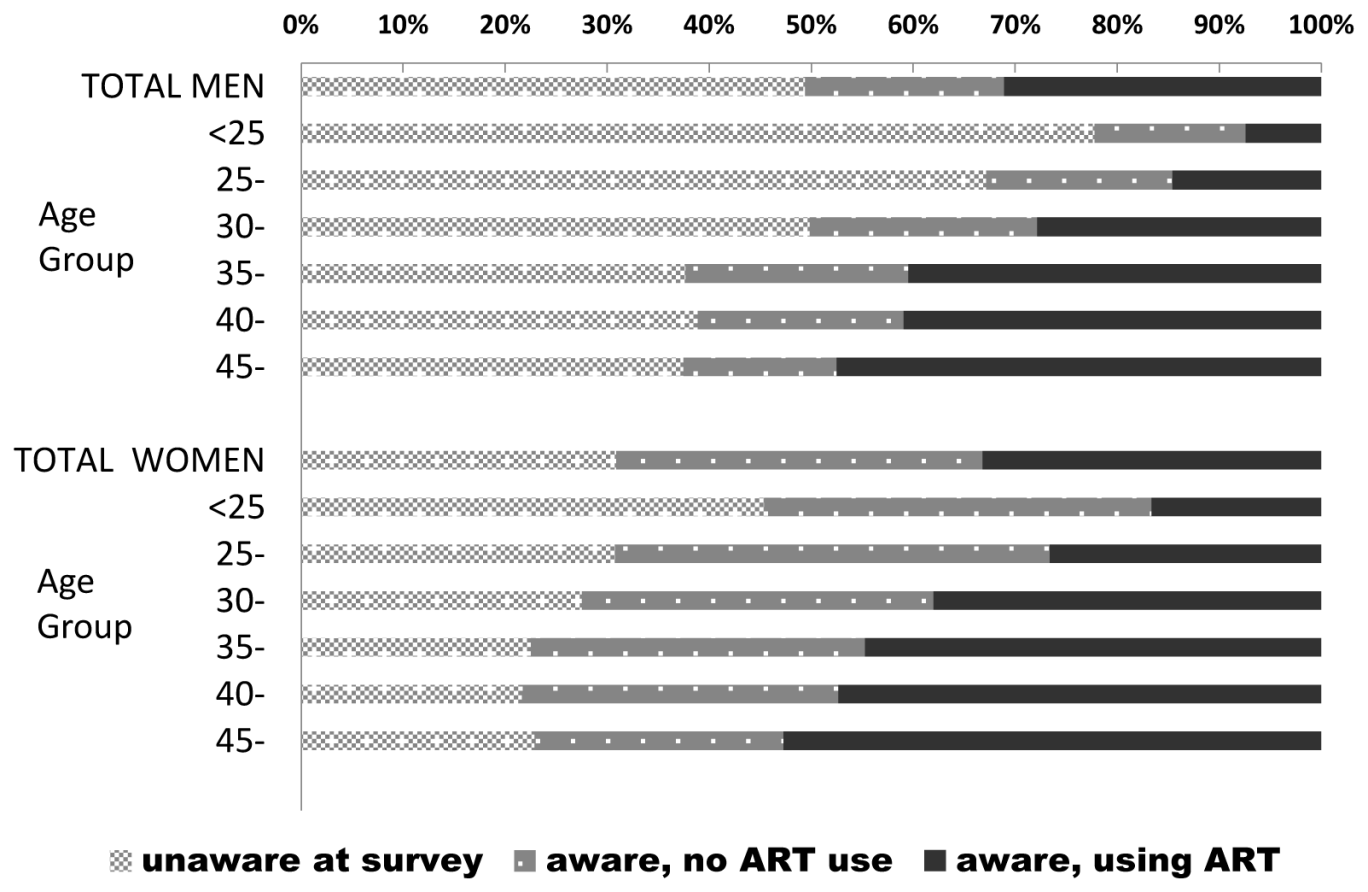
ART use and awareness of HIV status among HIV-infected individuals, by age and sex. SHIMS 2011.

HIV-infected men were far more likely (50%) than infected women (31%) to be unaware of their positive serostatus, but among those who were aware of their positive status, a larger proportion of men (60%) than women (46%) reported ART use (Figure 3). This prevailing pattern of service utilization results in nearly twice as many HIV infected women (35%) than men (19%) knowing their HIV positive status but not using ART. Age-related differentials in knowledge of HIV serostatus and HIV service utilization are striking. Nearly 4 in 5 (78%) HIV infected men under age 25 were not aware of their serostatus, compared to about one-third (38%) of infected men age 45+ years. Comparable figures for women were 46% and 23%, respectively. Of note, knowledge of HIV serostatus and ART use among the HIV-infected did not vary substantially between urban and rural areas in Swaziland (data not shown). 

## Discussion

In this cross-sectional survey of a household-based, nationally representative sample of adults (age 18-49) in Swaziland, HIV prevalence was 32%, representing the national adult HIV prevalence in 2011. The substantial inter-survey prevalence declines observed during 2006 to 2011 in the young age cohorts (particularly men) and prevalence rises in the older age groups (particularly women) suggest (1) a reduction in HIV incidence in the past four to five years, resulting in lower prevalence at young ages (fewer recent infections), and (2) improved survival among HIV positive people, resulting in higher prevalence in older age groups. These changing age-related patterns in HIV prevalence are not wholly unexpected. The past two rounds of the antenatal clinic-based HIV surveillance have shown declines in HIV prevalence among young pregnant women (ages 15-24) [8]. That prevalence rates for young men and non-pregnant women are here also displaying consistent downwards trends is encouraging and further support previous interpretation of reductions in new infections. 

Another key finding is that since the 2006-7 SDHS, gains have been made in the proportion of persons who have accessed HIV testing and counseling services (HTC) and are aware of their HIV status. In the SDHS 75% of men and 53% of women ages 18-49 reported having *never* been tested for HIV, compared to 45% and 16% in men and women, respectively, in SHIMS 2011. Despite the gains, a still substantial proportion of men (50%) and women (31%) with a positive HIV test in SHIMS were unaware of their HIV status at the time of their test. These findings highlight the need for a sustained effort to increase HTC frequency and coverage levels in the general population of Swaziland, with a particular emphasis on increasing access in men and improving linkage of both men and women to now widely available HIV prevention and care services. 

Swaziland has shown progress in responding to the growing need for ART, from 2004 when very few Swazis were receiving the benefit of ARVs to end-2011 when 81% of the eligible population (at CD4+ level below 350 cells/mm^3^) are estimated to have received ART through the national program [9]. A recent national ART program evaluation has underscored the importance of bringing ARVs closer to those in need through decentralization of care services [10]. Efforts to document expected declines in HIV-related mortality have not yet not been implemented. The rise observed here in HIV prevalence at ages above age 30 for women and age 35 for men is supportive evidence of improved survival as a result of ART. That this effect is especially pronounced in women, who represent 65% of adult ART users in the country, further bolsters this interpretation. Increases in HIV incidence in the older population, while a less parsimonious explanation, cannot be wholly discounted. 

The pattern of low/late uptake of HTC by men especially is consistent with late entry into care. Data from the National ART program evaluation demonstrate that men initiate ART at a lower CD4+ level (mean = 114) than women (mean = 158) and that the percentage of men who are already WHO stage 4 at initiation is almost double that amongst women (18% versus 10%) [10]. this emphasizes the need to strengthen programs targeting male involvement in HTC and subsequent linkage to care. 

While reduced prevalence and indications of falling infection rates in young persons indicate progress in slowing the epidemic, it is apparent that both young men and women (ages <25) are underutilizing HIV services. This has important implications for HIV prevention programs as youth represent a generally receptive target group for adoption of medical male circumcision and efforts to reduce early sexual debut. For youth already infected, timely entry into care services and positive prevention programs would be expected to have a long-lasting positive impact in reducing new infections and mitigating health and social costs. Given the very large proportion of HIV-infected males (78%) and females (46%) under 25 who do not know their status, it is recommended that efforts to increase the coverage and frequency of HIV testing and counseling in this group be intensified. 

The relatively high uptake of HTC services among women has resulted in a large pool of women who know they are HIV-positive but have not yet accessed care services. Current rates of linkage to care for Swaziland are not known, although current research in the country has this focus. The estimated retention rate of ART patients in HIV care at 12 months in Swaziland is 82% [10], which is above the median level of 75% found in a multi-country study of ART retention in sub-Saharan Africa [11]. In addition to HIV testing and care, establishing and maintaining one’s HIV-negative status through enhanced preventative services is also a principle objective of the Swaziland’s public health leadership. According to SHIMS, 61% of Swazi women and 76% of Swazi men age 18-49 are HIV-negative. In a country that continues to experience the highest national HIV prevalence in the world, our challenge remains to support uninfected men and women in accessing high quality prevention services , including voluntary medical male circumcision for men, HIV risk counseling, and widespread access to condoms.
